# Models of light absorption enhancement in perovskite solar cells by plasmonic nanoparticles

**DOI:** 10.1002/EXP.20220146

**Published:** 2023-09-06

**Authors:** Daming Zheng, Thierry Pauporté, Catherine Schwob, Laurent Coolen

**Affiliations:** ^1^ Sorbonne Université CNRS, Institut de NanoSciences de Paris, INSP Paris France; ^2^ Chimie ParisTech PSL Research University CNRS, Institut de Recherche de Chimie Paris (IRCP), Curie Paris France

**Keywords:** enhanced light absorption, perovskite solar cells, plasmonic nanoparticles

## Abstract

Numerous experiments have demonstrated improvements on the efficiency of perovskite solar cells by introducing plasmonic nanoparticles, however, the underlying mechanisms are still not clear: the particles may enhance light absorption and scattering, as well as charge separation and transfer, or the perovskite's crystalline quality. Eventually, it can still be debated whether unambiguous plasmonic increase of light absorption has indeed been achieved. Here, various optical models are employed to provide a physical understanding of the relevant parameters in plasmonic perovskite cells and the conditions under which light absorption may be enhanced by plasmonic mechanisms. By applying the recent generalized Mie theory to gold nanospheres in perovskite, it is shown that their plasmon resonance is conveniently located in the 650–800 nm wavelength range, where absorption enhancement is most needed. It is evaluated for which active layer thickness and nanoparticle concentration a significant enhancement can be expected. Finally, the experimental literature on plasmonic perovskite solar cells is analyzed in light of this theoretical description. It is estimated that only a tiny portion of these reports can be associated with light absorption and point out the importance of reporting the perovskite thickness and nanoparticle concentration in order to assess the presence of plasmonic effects.

## Graphical abstract

The experimental literature on plasmonic perovskite solar cells is analyzed in light of this theoretical description. It is estimated that only a small portion of these reports can be associated with light absorption and point out the importance of reporting the perovskite thickness and nanoparticle concentration in order to assess the presence of plasmonic effects like localized surface plasmon resonance.

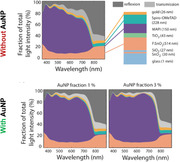

## 1 INTRODUCTION

Photovoltaic solar cells based on organic–inorganic lead halide perovskite semiconductors have emerged as one of the most promising technology in the field for both single and multijunction devices.^[^ ^1^, ^2^, ^3^ ^]^ These perovskite solar cells (PSC) have demonstrated high‐power conversion efficiencies which now exceed 25% for a single junction.^[^ ^4^, ^5^ ^]^ These remarkable achievements are attributed to perovskite exceptional properties, including high optical absorption, low exciton binding energies, long‐range charge carrier diffusion lengths and simple mild temperature processing from solutions.^[^ ^6^ ^]^ Moreover, the bandgap of the halide perovskite compounds can be easily tuned and optimized via the simple interchanges of their composition. Optimizing the perovskite layer for both solar light absorption and charge transport as well as the interfaces with the selective contact layers are important for the device optimization.^[^ ^7^, ^8^ ^]^

Light absorption in a solar cell can be improved by inserting plasmonic nanostructures.^[^ ^9^ ^]^ In particular, for solution‐processed cells such as PSCs, metallic nanoparticles (NP) can be introduced into the device's architecture with minor change to the fabrication protocol. For some metals such as gold or silver, these NPs present a localized surface plasmon resonance (LSPR), a coupled oscillation of the charges in the metal and of the local electromagnetic field. Two mechanisms may then benefit light absorption: (i) enhanced (LSPR) electromagnetic « near‐field » around the NP, and (ii) « far‐field » light scattering increasing the optical path travelled in the active layer.^[^ ^10^, ^11^, ^12^ ^]^

An increasing number of articles have described experimental plasmonic PSC realizations,^[^ ^13^, ^14^, ^15^, ^16^, ^17^, ^18^, ^19^, ^20^, ^21^, ^22^, ^23^, ^24^, ^25^, ^26^, ^27^, ^28^, ^29^, ^30^, ^31^, ^32^, ^33^, ^34^, ^35^, ^36^, ^37^, ^38^, ^39^, ^40^, ^41^, ^42^, ^43^, ^44^, ^45^, ^46^, ^47^, ^48^, ^49^, ^50^, ^51^, ^52^, ^53^, ^54^, ^55^, ^56^, ^57^, ^58^ ^]^ as reviewed in refs. [10–12, 59–67] Power conversion efficiency (PCE) improvements up to 60 % (*relative* increase) were reported in a few papers, however starting from relatively inefficient original cells. More generally, significant PCE improvements of the order of 2% (*absolute* increase) were obtained and correlated with an improvement of the short‐circuit current (see Supporting information Section C for a full analysis of the state of the art). However, some doubts remain about the reasons for these improvements and whether they can be attributed to better light absorption. Metallic NPs have also been reported to decrease exciton binding energy^[^ ^39^ ^]^ or benefit charge collection, as well as to improve perovskite growth^[^ ^11^, ^68^ ^]^ or stability.^[^ ^60^, ^67^ ^]^

Optical LSPR effects in PSCs have been modelled numerically by many authors and the role of the different NP properties has been discussed.^[^ ^69^, ^70^, ^71^, ^72^, ^73^, ^74^, ^75^, ^76^, ^77^, ^78^, ^79^, ^80^, ^81^, ^82^ ^]^ The simulations were used to analyze the role of the different parameters: NP composition, size, shape, aspect ratio, presence of a shell,^[^ ^70^, ^78^ ^]^ NP position in the cell stacking,^[^ ^75^, ^78^ ^]^ concentration,^[^ ^70^, ^71^, ^78^ ^]^ dependence on perovskite layer thickness,^[^ ^72^, ^78^ ^]^ role of thermal effects,^[^ ^81^ ^]^ etc. Various structures were recommended for light absorption optimization: Ag “lumpy” NPs in very thin 10‐nm perovskite layer,^[^ ^72^ ^]^ oblate Al NPs,^[^ ^77^ ^]^ 60‐nm radius Au NPs,^[^ ^73^ ^]^ Ag ellipsoidal NPs,^[^ ^81^ ^]^ Cu NPs,^[^ ^69^ ^]^ a combination of Ag and Al NPs,^[^ ^78^ ^]^ Al spherical NPs on top of the cell and within the active layer,^[^ ^75^ ^]^ closely‐spaced arrays of Ag NPs,^[^ ^76^ ^]^ TiN or ZnN NPs on top of the cell^[^ ^74^ ^]^ etc. However, the design and interpretation of plasmonic PSC experiments is still insufficiently guided by theoretical optical predictions.

The aim of this article is to provide simple models to point out the main physically relevant parameters for optical improvement in plasmonic PSCs and to use this perspective to discuss the recent literature experimental achievements. Theoretical works on plasmonic NPs in PSCs have generally used numerical simulations, by discrete‐dipole approximation,^[^ ^77^ ^]^ finite integral technique^[^ ^76^ ^]^ and mostly by finite difference methods.^[^ ^70^, ^71^, ^72^, ^73^, ^74^, ^75^, ^78^, ^81^ ^]^ Some authors have also developed analytical effective index models^[^ ^69^, ^83^ ^]^ or a combination of analytical and numerical models.^[^ ^79^, ^82^ ^]^ Although numerical simulation methods provide more flexibility, in this paper we have used analytical formalisms in order to provide a more physical understanding of the different effects and the role of approximations. All readers can easily implement these formalisms through fast open‐source codes.

In the first section, we describe the perovskite solar cell (without NP) in order to show which aspects need improvement. We then describe the NP LSPR properties (not in PSC) in order to show which NP properties could be adjusted to enhance light absorption. We start within the small‐NP approximation (Section 3), and then use the recent generalized Mie theory to analyze size effects for larger NPs (Section 4). Finally, in Section 5, we model a PSC where the perovskite contains Au NPs, evaluate the improvements which can be expected, and use our theoretical analysis to discuss the experimental state of the art and propose future directions.

## 2 PEROVSKITE SOLAR CELLS

### 2.1 Model

**FIGURE 1:**
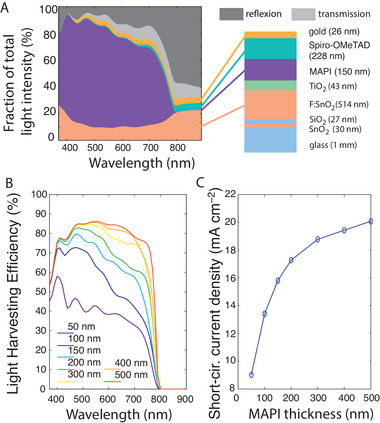
Optical model of the perovskite solar cell. (A) Fractions of incident light transmitted (light grey), reflected (dark grey) or absorbed (colours) by the various cell layers with 150‐nm MAPI thickness. Right: schematic of the cell. (B) Light harvesting efficiency (fraction of incident light absorbed by the active perovskite layer) for different MAPI thicknesses. (C) Corresponding short‐circuit current density (assuming perfect charge collection and extraction) as a function of the perovskite layer thickness. Figures (A) and (B) were obtained by the transfer‐matrix method.

Among hybrid organic‐inorganic perovskites used in solar cells, methylammonium lead iodide (MAPbI_3_, here labelled MAPI) has been explored earliest and most extensively. MAPI has been used in almost all studies on metallic NP doping in PSCs, so that we will limit our present discussion to this halide perovskite. A typical simple PSC device is represented in Figure 1A. In the literature, the front electrode is often a commercial wafer of transparent fluoride‐doped tin oxide (F:SnO_2_) on a glass substrate with two thin SnO_2_ and SiO_2_ bonding layers.^[^ ^84^ ^]^ Titanium dioxide (TiO_2_) is then deposited as an electron transport layer (ETL), MAPI as the active layer, and spiro‐OMeTAD for the hole transport layer (HTL). Finally, a gold layer serves as the back electrode. We used the optical indices reported previously in ref. [84] as plotted in the supporting information (Figure S1).

Light transmission, reflection and absorption by this device can be computed instantly by the transfer‐matrix method (TMM) which takes into account light reflection at each interface and absorption within each layer. We used the powerful commercial TMM software Film Wizard but open‐source TMM codes can be found online.^[^ ^85^ ^]^

### 2.2 Light absorption

Figure 1A shows the result for a device with a 150‐nm MAPI layer. Light absorption by MAPI is of course the dominant effect for wavelengths below the band gap (800 nm). Absorption by the front electrode (F:SnO_2_) is the main loss mechanism. Light reflection, in particular at the front electrode entrance (air‐glass interface), is another weaker loss mechanism. Minor portions of the incident light are also absorbed by spiro‐OMeTAD and gold or transmitted through the device.

The fraction of incident light that is absorbed by the active layer is defined as the light harvesting efficiency (LHE). Figure 1B plots the LHE spectrum for different MAPI thicknesses. The LHE is higher in the blue domain because MAPI absorption is stronger. The LHE increases significantly with the perovskite thickness, and saturates for thicknesses around 300–500 nm as the light reaching the MAPI layer is fully absorbed then.

The corresponding short‐circuit current under 100 mW cm^‐^^2^ illumination can be calculated, assuming that all generated electrons are collected by the electrodes without loss (Figure 1C). It increases dramatically with the MAPI thickness between 50 and 300 nm. For thicknesses above 300 nm, the LHE improvement is weaker and may be compensated by more crystalline defects and collection losses, so that typical devices choose thicknesses around 300–400 nm.

The 500‐nm‐layer case shows what can be obtained at most, when the MAPI layer is sufficiently thick to be fully absorbent. For a typical device with 300 to 400 nm active layer, the short‐circuit current is only 2–5% below this maximum: almost all of the light reaching the MAPI layer is already absorbed. The significant loss mechanisms in the typical device are light reflection and absorption by the front electrode, because they occur before light enters the active layer (see ref. [84] for a full discussion). They may be mitigated by procuring thinner front‐electrodes or of a different material and by using anti‐reflection coating at the front electrode entrance, but not by improving MAPI absorption.

### 2.3 Conclusions

The absorption of a MAPI layer of standard thickness of 300–400 nm is already optimal and can only be slightly increased. The main potential outcome of rendering the perovskite more absorbent by NP introduction is that it might allow to achieve the same photovoltaic efficiency but with a thinner layer. This may be of interest in the search for new systems (such as more stable perovskite materials, tandem silicon‐perovskite devices, and large‐scale industrial fabrications) if the use of a thick layer is then prevented by excessive crystalline defects.

As appears in Figure 1B, the need for absorption improvement is stronger in the red domain (600–800 nm), so this is the range where the LSPR resonance must be sought for.

## 3 LSPR IN THE “SMALL” NP LIMIT

We now turn to the LSPR modes of the metallic NPs, starting with the regime of small NPs, for which a simple analytical treatment is possible. This approximation will provide insight into the main physical mechanisms, and holds roughly for most experimental reports in the literature as the NP size is generally in the 15–50 nm range, with a few cases larger than 50 nm^[^ ^14^, ^24^, ^27^, ^28^, ^31^, ^33^, ^39^, ^41^, ^42^, ^43^, ^48^ ^]^ and fewer cases in the range 8–15 nm.^[^ ^19^, ^21^, ^30^, ^38^, ^46^, ^53^, ^57^ ^]^ In the next section, we will use Mie's theory to describe spherical NPs of any size and analyze size effects.

### 3.1 Model

**FIGURE 2:**
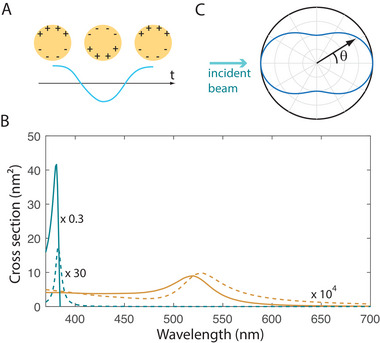
Plasmonic properties in the small nanoparticle regime. (A) Schematic of a LSP mode for a small NP. (B) Absorption and scattering spectra (resp. full and dotted lines) for a gold (yellow) or silver (blue) NP of radius 3 nm. (C) Scattering diagram of a small NP illuminated from the top.

For small particles (radius R≪λ), relatively simple expressions can be derived within Rayleigh's approximation.^[^ ^86^, ^87^ ^]^ The plasmonic oscillation then consists of a separation of negative charges on one side of the NP and positive charges (electron vacancies) on the other side (Figure 2A). It can be described by the induced dipole $\vec{p}=\;\varepsilon_{m}\alpha \vec{E_{0}}$ where α is the electrostatic polarizability and $\varepsilon_{m}$ is the medium permittivity. The absorption ($\sigma_{abs}$) and scattering ($\sigma_{sca}$) cross‐sections are given respectively by:

(1) $$\;\sigma_{abs}=\;\frac{2\pi }{\lambda }Im\left(\alpha \right)$$

and:

(2) $$\;\sigma_{sca}=\;\frac{8\pi^{3}}{3\lambda^{4}}{\left|\alpha \right|}^{2}$$

For a spherical NP of dielectric permittivity ε_1_ in a homogeneous environment of permittivity $\varepsilon_{m}$, the polarizability is:

(3) $$\alpha \left(\lambda \right)\;=\;4\pi R^{3}\frac{\varepsilon_{1}\left(\lambda \right)-\;\varepsilon_{m}\left(\lambda \right)}{\varepsilon_{1}\left(\lambda \right)+2\varepsilon_{m}\left(\lambda \right)}$$

### 3.2 LPSR wavelength

The absorption and scattering spectra $\sigma_{abs}\left(\lambda \right)$ and $\sigma_{sca}\left(\lambda \right)$ (Figure 2B) may thus exhibit a resonance peak, depending on the NP material and its environment optical index. The localized surface plasmon (LSP) absorption and scattering wavelengths, defined as the respective maxima of these spectra, are slightly different from one another. They can be approximated by the LSPR Fröhlich wavelength, which is the wavelength at which the real part of the denominator $\varepsilon_{1}+2\varepsilon_{m}$ vanishes.

It is critical to note in the polarizability expression that the LSPR wavelength depends, not only on the NP optical index, but also on the optical index of its environment. In fact, a slight change of the environment's index is sufficient to induce a significant spectral shift of the plasmon: this is how metallic surfaces or NPs are used as surface plasmon resonance (SPR) chemical or biological sensors. Therefore, in the context of NP‐doped solar cells, the LSPR wavelength will not be the same depending on which material it is inserted in. Table 1 gives the absorption and scattering wavelengths for spherical NPs in a few different materials relevant to PSC.

**TABLE 1:** Absorption and scattering wavelengths (defined as the respective maxima of $\sigma_{abs}\left(\lambda \right)$ and $\sigma_{sca}\left(\lambda \right)$) and Fröhlich wavelength (defined by $Re(\varepsilon_{1}+2\varepsilon_{m})\;=\;0$), for a small NP of gold or silver in different environments.

	Medium	*λ_F_* (nm)	*λ_abs_* (nm)	*λ_sca_* (nm)
Au NP	Water	513	520	529
	Glass	526	527	533
	MAPI	640	649	646
	Spiro.	533	535	540
	TiO_2_	602	604	603
	F:SnO_2_	560	561	564
Ag NP	Water	383	383	383
	Glass	401	398	398
	MAPI	565	583	580
	Spiro.	426	426	426
	TiO_2_	514	514	514
	F:SnO_2_	457	457	457

### 3.3 LSPR absorption and scattering

The dependence on the NP radius is given by the*R* ^3^term in the polarizability expression: the absorption cross‐section will be proportional to the NP's volume, while the scattering cross‐section will scale as *R* ^6^. Therefore, for small NPs (as long as R≪λ), scattering is negligible compared to absorption.

Away from the LSP resonance, the absorption cross section scales roughly as 1/λ while $\sigma_{sca}\left(\lambda \right)$ shows the well‐known Rayleigh scattering dependence $\lambda^{-4}$.

The angular distribution of the light intensity scattered by a particle is expressed by the scattering diagram (also called phase function) ^[^ ^87^ ^]^ $p\;\left(\theta ,\varphi \right)=\;\left(dP_{sca}/\sin\theta \;d\theta \;d\varphi \right)/P_{sca}$. In the limit of a small NP, the scattering diagram for unpolarized light is:

(4) $$p\left(\theta \right)=\frac{3}{16\pi }\left(1+\cos^{2}\theta \right)$$

where θ is the angle between the incident and the scattered beams.^[^ ^87^ ^]^ This diagram presents a peanut shape (Figure 2C) with maximal scattering into the forward (θ=0) and backward (θ=π) directions, but also significant scattering into other directions.

### 3.4 Ellipsoidal particles

Ellipsoidal particles are among the few non‐spherical geometries for which polarizability can be expressed analytically.^[^ ^87^ ^]^ They can be used to understand the behaviour of elongated plasmonic nanorods, even though these may be closer to cylinders than ellipsoids. Along the ellipsoid's eigenaxis i(i=x,y,z), the polarizability is expressed as:

(5) $$\;\alpha_{i}=\;4\pi R_{x}R_{y}R_{z}\;\frac{\varepsilon_{1}-\varepsilon_{m}}{3\varepsilon_{m}+3L_{i}\left(\varepsilon_{1}-\varepsilon_{m}\right)}$$

with:

(6) $$\begin{array}{ccc} & & \;L_{i}\\ & & =\;\frac{R_{x}R_{y}R_{z}}{2}\int_{0}^{\infty }\frac{dq}{\left({R_{i}}^{2}+q\right)\sqrt{\left(q+{R_{x}}^{2}\right)\left(q+{R_{y}}^{2}\right)\left(q+{R_{z}}^{2}\right)}} \end{array}$$

**FIGURE 3:**
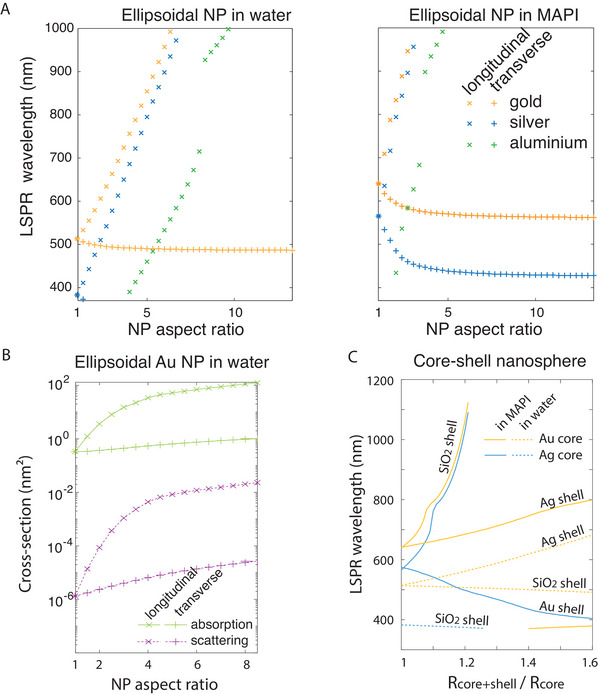
Plasmonic properties of ellipsoidal or core‐shell structures in the small‐NP regime. (A) Fröhlich LSPR wavelength of a gold, silver or aluminium ellipsoidal nanoparticle in a water (left) or MAPI (right) surrounding medium, as a function of its aspect ratio, for an electric field either parallel to the particle long or short axis (respectively longitudinal and transverse LSP modes). (B) Absorption (full lines) and scattering (dotted lines) of the LSPR modes of an elliptical gold NP in water, as a function of its aspect ratio, the radius of the NP along the transverse axes being 1 nm. (C) LSPR Fröhlich wavelength of a gold (yellow lines) or silver (blue lines) nanosphere coated by a silica, silver or gold shell, in a water (dotted lines) or MAPI (full lines) medium.

Figure 3 plots the ellipsoid's Fröhlich wavelengths (now defined by $Re(3\varepsilon_{m}+3L_{i}\left(\varepsilon_{1}-\varepsilon_{m}\right))\;=\;0$) as a function of the its aspect ratio in water and in MAPI (Figure 3A). For the LSP modes polarized along the particle's short axes (transverse modes), the wavelength is blue shifted by only a few tens of nanometres. The long‐axis LSP mode (longitudinal mode), on the other hand, depends strongly on the particle's aspect ratio, so that its wavelength can be redshifted by hundreds of nanometres. Using metallic nanorods instead of nanospheres thus provides a way to tune the plasmonic resonance, although only for one polarization of the incident light. For this longitudinal polarization, the absorption cross‐section is also strongly enhanced as the NP aspect ratio increases (Figure 3B), while it shows little variation for the transverse modes. The scattering cross‐section is also much higher for the elongated mode than for the transverse mode. It always remains negligible as compared to the absorption cross‐section as we are within the small‐NP approximation.

**FIGURE 4:**
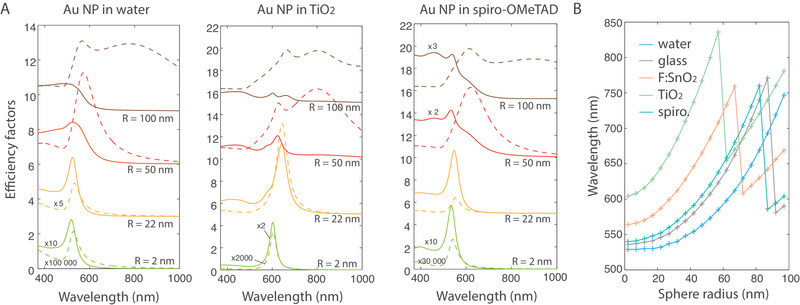
Spectral modifications as a function of the NP size in various dielectric environments. (A) Absorption (full lines) and scattering (dotted lines) efficiency factors of a gold nanosphere of radius 2, 22, 50 or 100 nm, in a water, TiO_2_ and spiro‐OMeTAD medium, as calculated by Mie theory. The efficiency factors $Q_{abs}$ and $Q_{sca}$ are defined respectively as $\sigma_{abs}/\pi R^{2}$ and $\sigma_{sca}/\pi R^{2}$.^[^ ^87^ ^]^ (B) LSPR wavelength (estimated as the maximum of the scattering spectrum because no absorption maximum exists for high radii) as a function of the sphere radius, in different environments.

Most experiments in the literature have used gold or silver particles, so that LSPR wavelengths were reported respectively in the ranges 520–560 and 400–450 nm. It must be noted however that the authors always measured these wavelengths by absorption spectroscopy in a solvent (typically ethanol) whose index is very different from the index of the perovskites. As shown previously (Table 1, Figure 4), the LSPR wavelength should thus be very different inside the PSC from its measured value in solution. This point is discussed in theoretical papers but very few authors of experimental work mention this effect.^[^ ^21^, ^26^, ^54^ ^]^

As a consequence, LPSR effects are often anticipated incorrectly in the blue‐green domain. In order to shift the LSPR to the more critical red domain, various authors have introduced gold nanorods. Indeed, the additional longitudinal LPSR lies in the range 660–790 nm^[^ ^26^, ^34^, ^36^, ^44^, ^45^ ^]^ for the nanorods in solution. However, when inserted into the perovskite, the LSPR shifts very quickly beyond the band gap (Figure 3A), so that this strategy is in fact shifting the LSPR away from the relevant spectral range.

A more interesting use of non‐spherical shapes is to replace the spherical NPs by sharper geometries such as nanocubes,^[^ ^25^, ^28^, ^41^ ^]^ nano‐octahedrons,^[^ ^33^ ^]^ nanorods,^[^ ^26^, ^27^, ^34^, ^36^, ^44^, ^45^ ^]^ popcorn nanoparticles,^[^ ^43^ ^]^ nanobipyramids,^[^ ^15^ ^]^ nanostars,^[^ ^18^, ^37^, ^50^ ^]^ crescent‐shaped NPs^[^ ^24^ ^]^ or NP dimers.^[^ ^27^ ^]^ Confined LSP modes are then anticipated at the NP's tips with much stronger electromagnetic fields leading to much improved light absorption.

### 3.5 Core–shell NP

In reported PSC experiments, a shell of a different material,^[^ ^21^, ^22^, ^23^, ^29^, ^33^, ^36^, ^40^, ^51^, ^53^ ^]^ typically silica,^[^ ^25^, ^26^, ^27^, ^28^, ^39^, ^45^, ^46^, ^47^, ^49^, ^52^, ^56^ ^]^ was often added to the NPs, in order to improve their chemical and thermal stability^[^ ^36^, ^39^, ^49^, ^52^ ^]^ and protect them from corrosion by the perovskite^[^ ^27^, ^28^, ^30^, ^47^, ^51^, ^56^ ^]^ as well as to avoid exciton recombination at the NP surface.^[^ ^25^, ^36^, ^45^, ^52^, ^56^ ^]^When remaining in the small‐NP limit, the polarizability of such NPs can be calculated analytically^[^ ^87^ ^]^ and their Fröhlich wavelength satisfies:

(7) $$\left(\varepsilon_{2}+2\varepsilon_{m}\right)\left(\varepsilon_{1}+2\varepsilon_{2}\right)+2f\left(\varepsilon_{2}-\varepsilon_{m}\right)\;\left(\varepsilon_{1}-\varepsilon_{2}\right)=\;0$$

where ε_2_ is the shell material permittivity and $f\;=\;{\left(R_{core+shell}/R_{core}\right)}^{3}$ is the ratio between the core + shellvolume and the core volume. Figure 3C plots the LSPR Fröhlich wavelength as a function of the ratio $R_{core+shell}/R_{core}$ for several core, shell and environment materials. The spectral shift induced by the shell can be very large. Even when the shell thickness is only 10% of the core radius, a shift of a few tens or even hundreds of nm is calculated. The choice of NP coating, usually motivated by chemical or electrical reasons, must thus also take into account the LSPR shift induced by the shell. Moreover, numerical simulations have shown that the silica shell thickness must remain below around 10 nm to maintain the near‐field absorption enhancement.^[^ ^70^, ^78^ ^]^

### 3.6 Conclusions

Simple analytical equations can describe the LSPR properties of small NP and the role of the different parameters: optical index of the NP and its environment, aspect ratio for a non‐spherical NP, presence of a shell etc. The LSP resonance wavelength depends strongly on the optical index of the surrounding medium, which is much higher in MAPI than in typical solvents, so that the LSPR wavelength in MAPI can actually not be known from standard cuvette measurements. For small gold NPs, the LSPR is conveniently located around 640 nm, where MAPI absorption needs to be enhanced. It shifts when a protective dielectric shell is added.

## 4 LPSR: MIE THEORY

In order to model larger NP and understand size effects, the more complex Mie equations must be used.

### 4.1 Model based on Mie theory

The problem of light absorption and scattering by a spherical particle of arbitrary (not ≪λ) size can be solved exactly by a formalism usually referred to as Mie theory.^[^ ^88^ ^]^ The resulting equations are quite cumbersome but can be computed quickly. We used the open‐source code originally published by Bohren and Huffman.^[^ ^89^ ^]^

Mie theory can be applied only to particles inside a transparent medium. However, when introducing metallic NPs in the active layer of a solar cell, the NP's surrounding is necessarily absorbing. There is no simple way to adapt Mie's formalism to a NP in an absorbing environment.^[^ ^90^ ^]^ Different definitions could be chosen for the absorption and scattering cross‐sections, either from the near‐field or far‐field points of view.^[^ ^91^, ^92^, ^93^, ^94^ ^]^

To our knowledge, very few authors have discussed the effect of an absorbing environment on the Mie resonances in the context of photovoltaics,^[^ ^95^, ^96^ ^]^ and none for perovskite cells. We use here the far‐field “generalized Mie” formalism, developed by M. I. Mishchenko in 2007 in the context of atmospheric spectroscopy^[^ ^97^ ^]^ and implemented in 2018 as an open‐source FORTRAN code^[^ ^98^, ^99^ ^]^.

### 4.2 LPSR spectra

**FIGURE 5:**
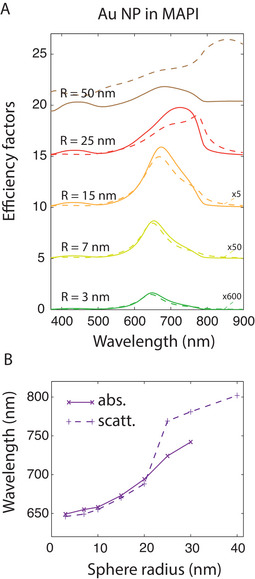
Spectral modifications as a function of the NP size in MAPI. (A) Absorption (full lines) and scattering (dotted lines) efficiency factors of a gold nanosphere of radius 2, 22, 50 or 100 nm, in a MAPI environment, as calculated by the generalized Mie theory. The efficiency factors $Q_{abs}$ and $Q_{sca}$ are defined as $\sigma_{abs}/\pi R^{2}$ and $\sigma_{sca}/\pi R^{2}$ respectively.^[^ ^87^ ^]^ (B) LSPR wavelength as a function of the sphere radius, estimated as the maximum of the absorption (full line) or scattering spectrum (dotted line).

We plot the absorption and scattering spectra of gold NPs of various radii in water, TiO_2_ and spiro‐OMeTAD (Mie theory—Figure 4A) and in MAPI (generalized Mie theory, Figure 5A)—absorption and scattering spectra of other radii for gold, silver and aluminium NPs can be found in the supporting material (Figures S2–S7). As noted in the case of small NPs, the LSPR wavelength is different depending on the surrounding environment. The resonance peak is sharp in TiO_2_ due to the higher index contrast between gold and titanium dioxide. On the other hand, the LSPR mode is much broader in MAPI because its quality factor is decreased in a lossy environment.^[^ ^100^ ^]^ For a 2‐nm radius, the spectra match the small‐NP formulas, with the LSPR peak near the Fröhlich wavelength, added to a $\sigma_{abs}\;\propto \;\lambda^{-1}$ and $\sigma_{sca}\;\propto \;\lambda^{-4}$ Rayleigh contribution. As the radius is increased, the LSPR shifts to longer wavelengths (as plotted in Figure 4B). For 50 or 100 nm spheres, the absorption and scattering spectra become broader due to the appearance of multipole resonant modes.

For the smaller radii (R= 3 nm), a broad peak is obtained around 650 nm for the gold NPs, 580 nm for the silver NPs and 400 nm for the aluminium NPs. The peak redshifts and broadens as the NP radius increases (Figure 5A,B). Gold nanospheres are thus perfectly suited spectrally to enhance the absorption of MAPI, which is weaker in the range 600–800 nm as explained in Section 2. The spectra for Ag and Al NPs are displayed in Figures S3–S6. For the silver NPs, this range is covered only for the larger radii. For the aluminium NPs, the LSPR always remain mostly below 600 nm with a maximum in the blue range and suggests no interest for MAPI engineering.

### 4.3 Absorption and scattering cross‐sections

**FIGURE 6:**
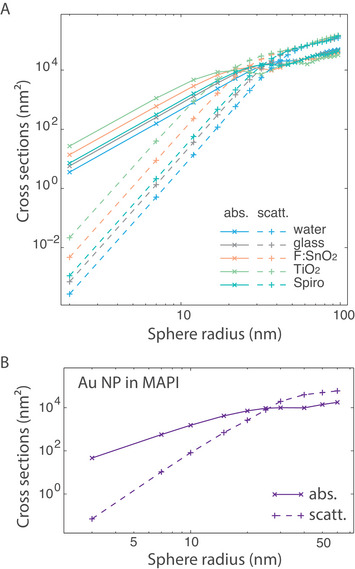
Absorption (full lines) and scattering (dotted lines) cross sections of a gold nanosphere as a function of its radius, (A) in water, glass, F:SnO_2_, TiO_2_ and spiro‐OMeTAD (given by Mie theory) and (B) in MAPI (calculated by the generalized Mie theory) (curves in log‐log scale).

Figure 6 shows the calculated absorption and scattering cross‐sections as a function of the NP radius, for a gold NP in water, glass, F:SnO_2_, TiO_2_ and spiro‐OMeTAD (Figure 6A, Mie theory) and in MAPI (Figure 6B, generalized Mie theory). For a NP radius up to 10–30 nm, $\sigma_{abs}$ and $\sigma_{sca}$ scale respectively as *R* ^3^ and *R* ^6^, in agreement with the small‐NP Eqs. ((1), (2), (3)). For larger radii, the two cross‐sections keep increasing with *R* but with a slower dependence. The $\sigma_{sca}/\sigma_{abs}$ ratio is at most 0.03 for NP radii below 7 nm, reaches unity at R=20–40 nm depending on which material surrounds the NP, and stabilizes around a factor of 2–5 for larger particles. The effect of scattering can thus be neglected only for nanoparticles smaller than 10–15 nm.

At fixed NP concentration (number of NP per volume unit), evidently both absorption and scattering will increase with NP size. What happens at fixed volume fraction (ratio of NP volume over total volume) is less obvious. Because the absorption cross‐section of each NP is proportional to its volume for R≤10−30 nm, at fixed volume ratio, the absorption will not depend on the NP size. For larger NP sizes, because $\sigma_{abs}$ increases slower than *R* ^3^, absorption will in fact decrease with increasing *R*. As for scattering, at fixed NP volume fraction, it will increase with the NP size for R≤10−30 nm because $\sigma_{sca}$ scales as *R* ^6^, and decrease for the larger NPs because $\sigma_{sca}$ increases slower than *R* ^3^.

### 4.4 Light scattering

**FIGURE 7:**
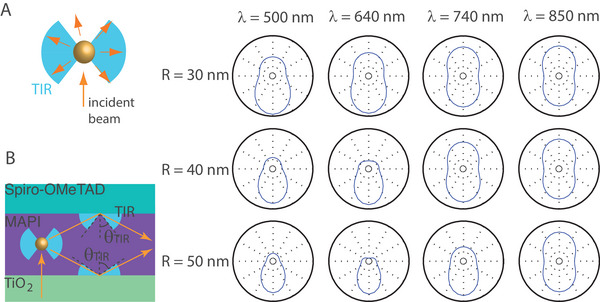
Scattering properties in MAPI. (A) Scattering diagram (arb. units) of a gold nanosphere in MAPI, as calculated by the generalized Mie theory, for various wavelengths and sphere radii. (B) Schematic of the total internal reflection (TIR) angles within the PSC active layer. The light blue shade indicates the propagation angles which will remain guided inside the MAPI layer.

The scattering diagram is shown in Figure 7A for a gold NP in MAPI and in Figures S8 and S9 for a NP in water, TiO_2_ or spiro‐OMeTAD. For a radius of a few nm, the peanut‐shaped scattering diagram is independent on the wavelength and given by the small‐NP approximation (Figure 2C). For R=30 nm (Figure 7A), the diagram is still similar to Rayleigh scattering in the red domain, because the small‐NP (R≪λ) is still valid, and the dipolar LSP resonance still dominates. On the other hand, at shorter wavelengths in the blue domain, backward‐scattering becomes significantly more efficient than forward. At R= 50 nm, where the scattering spectrum is broad and strongly multimode, the scattering diagram is directed predominantly backwards over the whole visible spectrum.

It has been argued that backward scattering can be useful to cause light to pass again in the active layer. However, this role is already fulfilled by the gold electrode, and it seems not clear whether NP back‐scattering within the MAPI layer (or the spiro‐OMeTAD layer) can be advantageous. If plasmonic NPs are introduced as light scatterers, a more relevant aim should be to redistribute light propagation to higher angles in order to increase the optical path length inside the active layer and hence increase light absorption. In particular, because the real component of the optical index is higher in the MAPI layer than in its upper and lower layers, light propagation can be guided within the active layer: beams above the total internal reflection (TIR) angle will be fully reflected (Figure 7B). At the MAPI‐TiO_2_ and MAPI‐spiro‐OMeTAD interfaces, the TIR angle is respectively $\theta_{TIR}=\;$60° and 37°. Relevant light scatterers in MAPI should thus scatter light to angles in the interval $\left[\theta_{TIR};\;\pi -\;\theta_{TIR}\right]$. This corresponds respectively to 41–73 % of the radiation diagram for the Rayleigh scattering (Figure 2C) and rather similar percentages for R= 50 nm, although the scattering diagram is different. Therefore, all NP radii provide similar advantages in terms of the redistribution to high angles, and larger NPs should be favoured for their higher scattering cross‐sections.

### 4.5 Conclusions

We reported here the first application of the generalized Mie theory to a NP in a perovskite medium. Due to the large index of MAPI, the LSPR wavelength is strongly redshifted with respect to its value in solvent, as obtained already in section III for small NPs. The generalized Mie theory shows that the LSPR spectrum is also broader in an absorbing medium. The LSPR mode of a gold nanosphere is found in the range 600–800 nm, where MAPI absorption needs to be most amplified, so that these particles appear the most fitting for plasmonic PSC. Silver NP present less ohmic losses but their spectral range is less interesting, and aluminium NPs present no resonance in the target spectral range. Eventually, gold NPs should be favoured for PSC optimization.

For NP radii below 10–20 nm, the dominant LSPR mechanism is light absorption, in relation with the near‐field enhancement of the electromagnetic field. Light absorption will be proportional to the NP volume fraction for small NPs and decrease for larger NPs. Light scattering becomes more significant at higher radii and is higher than absorption by a factor 2–5 above 40 nm. It may then redirect part of the light to propagation directions guided inside the active layer.

## 5 SOLAR CELLS WITH AUNP‐DOPED PEROVSKITE

We now assess the optical performance of a perovskite solar cell doped with gold nanoparticles.

### 5.1 Model

In the limit of small NP, it is possible to use the TMM as in section II, with the AuNP‐doped perovskite layer described as a single medium with an effective optical index describing both the perovksite and the AuNP. Various effective medium approximation formalisms have been developed in order to describe a mixture of two different materials by a single optical index. In particular, the Maxwell‐Garnett approximation describes a medium of permittivity $\varepsilon_{m}$ doped with small inclusions of permittivity ε_1_ as a homogeneous effective medium.^[^ ^87^ ^]^ For spherical inclusions, the effective dielectric permittivity is written:

(8) $$\;\varepsilon_{av}=\;\varepsilon_{m}\left[1+\;\frac{3f\left(\frac{\varepsilon_{1}\left(\lambda \right)-\;\varepsilon_{m}\left(\lambda \right)}{\varepsilon_{1}\left(\lambda \right)+2\varepsilon_{m}\left(\lambda \right)}\right)}{1-f\left(\frac{\varepsilon_{1}\left(\lambda \right)-\;\varepsilon_{m}\left(\lambda \right)}{\varepsilon_{1}\left(\lambda \right)+2\varepsilon_{m}\left(\lambda \right)}\right)}\right]$$

where *f* is the inclusion volume fraction.

Note that the dependence on the nanosphere radius is not described by this equation because it is valid only in the limit R≪λ. For particles larger than a few nanometres, size effects may be described by resorting to the extended Maxwell–Garnett–Mie, as described for a few photovoltaic materials in ref. [83] The treatment of ellipsoidal particles is also analytically feasible within the Maxwell‐Garnett formalism, but the resulting effective index will not differ from the case of spherical particles up to the second order in $\left(\varepsilon_{1}-\varepsilon_{m}\right)/\varepsilon_{m}$.^[^ ^87^ ^]^

The effective medium approximation is also unable to describe scattering effects, which might trap part of the light within the active layer and improve its absorption. However, this is not a problem as long as it is understood that this approximation concerns small NPs, for which scattering is anyway negligible as compared to absorption. For larger NPs, finite‐difference simulations would be necessary to include scattering contribution, at the cost of a very fine mesh to describe the confined plasmon modes and long computation times. Moreover, as scattering is expected to enhance the optical path inside the active layer, large cell lateral dimensions would need to be simulated. Radiative transfer equations may provide an interesting alternative formalism.

### 5.2 Enhanced light absorption

**FIGURE 8:**
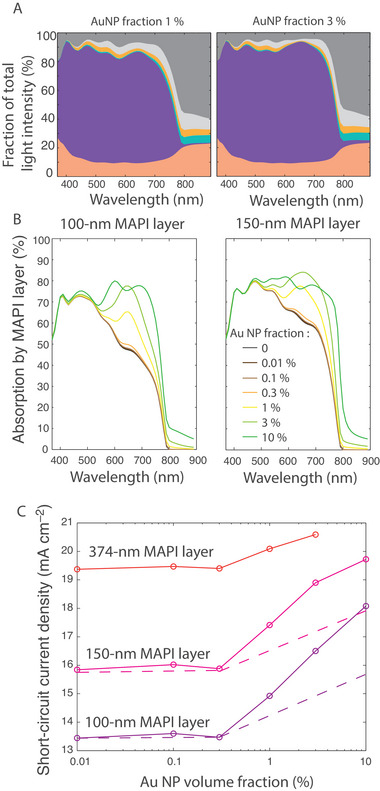
Solar cell properties with MAPI doped by Au‐NPs. (A) Fractions of incident light transmitted (light grey), reflected (dark grey) or absorbed (colours) by the various cell layers for a 150‐nm MAPI layer including a 1% (left) and 3% (right) volume fraction of gold nanospheres. (B) Spectrum of the fraction of light absorbed by the MAPI layer of either 100‐nm (left) or 150‐nm thickness (right) for several Au nanoparticles volume fractions. (A) and (B) were obtained by a transfer‐matrix method with the gold particles inclusion described with a Maxwell–Garnett effective medium approximation, therefore the results are only valid for small AuNP (R≪λ). (C) Corresponding short‐circuit current density (*J_sc_*) calculated as a function of the MAPI layer thickness and Au nanoparticles volume fraction, assuming that each absorbed photon leads to an electron collection. The dotted line shows what can be anticipated if we take into account that around half of the additional light absorption corresponds in fact to ohmic losses.

A PSC with a MAPI layer doped with small (R≪λ) AuNP (hereafter referred to as AuNP:MAPI) can thus be modelled by the transfer‐matrix method where the index of the AuNP:MAPI layer is obtained within the Maxwell–Garnett approximation. The distribution of light reflection, transmission and absorption is plotted in Figure 8A for a 150‐nm MAPI layer with gold‐NP volume fractions of 1% and 3%. Light reflection and absorption by F:SnO_2_ show little modification with respect to the case without gold NP (Figure 1C) because they occur before light reaches the MAPI layer. Absorption by the AuNP:MAPI layer is increased markedly in the red domain, as expected since it is the spectral range where MAPI absorption is not complete and where the LSP resonance occurs. Consequently, light transmission and absorption by gold and spiro‐OMeTAD are reduced. Some absorption by the MAPI layer appears above 800 nm: it corresponds however only to absorption by the NPs as light absorption by MAPI is negligible above 800 nm and cannot be enhanced.

Figure 8B plots the portion of incident light which is absorbed by a 100‐nm or 150‐nm Au‐doped MAPI layer, for different AuNP volume fractions up to 10% (although a 10% volume fraction may not be experimentally realistic as it should start to create excessive electric losses and crystalline defects; the effective medium approximation also begins to loose validity at such volume fractions as NP‐NP interactions should be taken into account^[^ ^83^ ^]^). Light absorption by the AuNP:MAPI layer shows very little change for volume factions up to 0.3%, then increases in the red domain as the volume fraction is increased to 1–3%, and does not change significantly above 3%.

In the literature, the NP volume fraction is usually estimated from the ratio of NPs and perovskite precursors in the spin‐coated solution. From the published data, we extrapolate that relevant fractions of 0.5%,^[^ ^27^ ^]^ 1%^[^ ^28^ ^]^ and 0.07%^[^ ^29^ ^]^ were used for NPs in the perovskite layer. For NPs deposited at the interface between two layers, the relevant parameter would be the NP surface concentration, but it is difficult to assess from the employed deposition protocol and is usually not reported.

In order to estimate which gain may be induced by this additional absorption, we used the MAPI layer absorption in Figure 8B and calculated the short‐circuit current, assuming that each photon absorption leads to an electron collection (which is not the case, as shown in the next subsection). For a 3% volume fraction, the current is increased by around 20% for a 100‐ or 150‐nm thick active layer, but only 7% for a 374‐nm thick layer. As explained in Section 2, the 374‐nm layer is already sufficiently thick to absorb almost all of the light reaching it, so that little improvement can be expected. On the other hand, for a thinner layer, significant improvement is possible. In particular, the 150‐nm layer with 3% gold fraction can reach the same short‐circuit current as the 374‐nm layer without gold NP.

The active layer thickness is thus a crucial parameter. However, we have found in the literature that this parameter is never reported or discussed in experimental papers. Moreover, it is never checked whether the perovskite thickness is the same with and without NPs. Based on the published supporting SEM images in some studies,^[^ ^29^, ^30^ ^]^ NP insertion may affect the thickness of the perovskite layer. This would lead indirectly to enhanced light absorption, but by a rather trivial mechanism, because an alternative strategy would be to use no NP and just deposit a thicker active layer.

### 5.3 Light absorption by gold versus MAPI

The short‐circuit current plotted in Figure 8C was calculated under the hypothesis that each photon absorbed by the active layer creates an electron contributing to the photovoltaic current. However, not all absorbed photons lead to charge creation in MAPI: one part of the absorption occurs in MAPI and is enhanced by the LSPR near‐field, while another part of the absorption occurs in gold and should be lost as heat dissipation.

The effective medium approximation does not allow us to estimate which portion of light is absorbed in gold and which is absorbed in MAPI. A few papers have discussed this question for metallic NPs in organic solar cells, analytically^[^ ^95^ ^]^ or numerically.^[^ ^96^ ^]^ For the moment, to our knowledge, no code has been published to apply the generalized Mie theory to distinguish absorption in the particle and in the surrounding medium. We thus resorted to a numerical finite‐difference calculation, using the commercial software Lumerical. As shown in the supporting information, it is crucial for such simulations to use a sufficiently fine mesh grid and to properly define the interface between the NP and its surrounding. Eventually, a simulation of a 7‐nm gold NP in a 100 × 100 × 100 nm^3^ MAPI cube, as compared to the same cube without NP, yields an additional 8% absorption. Half of this additional absorption occurs in the volume of the NP (heat losses) and half occurs in MAPI (useful absorption). For different radii from 3 to 15 nm, the absorption varies but the ratio between losses and useful absorption remains the same. Therefore, the actual increase of the short‐circuit current (*J_sc_*) due to the gold NP should be half of what is plotted in Figure 8C: we add it as a dotted line on Figure 8C. We find then that, even with AuNP inclusion up to 10% volume fraction, the 150‐nm thick composite layer cannot equal the absorption of the 374‐nm layer without AuNP.

### 5.4 Experimental evidence of light absorption enhancement

The results of the theoretical model can also be used to understand which experimental observations are a sign of improved light absorption. Indeed, the general observation of increased PCE and short‐circuit current (see Supporting Information Section C for a full literature analysis) may be attributed either to enhanced light absorption, or to electrical effects such as transport and collection improvements, suppression of charge recombination pathways or more efficient exciton dissociation.

**FIGURE 9:**
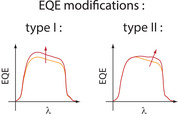
Schematic of two different types of external quantum efficiency (EQE) spectrum modifications : type I (improvement over the whole spectrum) suggests better charge separation or transport and type II (improvement mostly at wavelengths before the band gap) suggests increased light absorption.

The external quantum efficiency (EQE—ratio between the number of electrons collected and the number of incident photons) is a key element in order to distinguish between optical and electrical effects. Figure 9 shows two typical modifications of the EQE spectrum, both corresponding to a general increase of the PCE.

In the second case (type II), the EQE increase is stronger in the red domain, just before the bang gap. This case can be considered as a sign of improved light absorption by the active layer (see Figure 8B): because the perovskite absorbs less in the red domain, the EQE is more improved at these wavelengths. In the blue domain, the EQE is not increased because light absorption was already maximal.

In the first case (type I), on the other hand, the EQE increase is rather similar for all absorbed wavelengths: this is a sign that improved PCE should not be explained by a better light absorption.

Let us point out that the presence of LSPR effects should not manifest as clear sharp spectral features in the EQE spectrum, because the LSPR spectrum is much broader in an absorbing medium such as a perovskite (Figure 5A) than in a transparent medium such as a solvent.

Upon reviewing the literature, we have found that most of the reported EQE spectra fall within the type I class, so that the improved performance should be attributed to electrical effects. Some studies^[^ ^31^, ^35^, ^41^, ^42^, ^58^ ^]^ show a type II EQE modification indicative of enhanced light absorption. More knowledge of the experimental parameters (perovskite layer thickness etc.) and theoretical modelling would be necessary in order to understand precisely the mechanism for each of these reports. Various optical LSPR effects are possible: near‐field enhancement of the electromagnetic field, light scattering leading to longer optical path.

We note also that the quality of the perovskite layer may be modified when introducing the NPs: improvements of the crystalline structure, grain size and smoothness have been mentioned in refs. [20, 30, 31, 33] A careful analysis of the supporting data of other papers shows improvements in some SEM images^[^ ^15^, ^36^, ^38^, ^48^, ^55^, ^58^ ^]^ and XRD data^[^ ^35^, ^55^ ^]^ but NP addition also seems detrimental in some other cases.^[^ ^16^, ^17^, ^22^ ^]^ In our recent paper^[^ ^68^ ^]^, we found type‐II EQE improvements when introducing Au‐NP inside the perovskite layer. The volume fraction (0.01%) was much too low to induce LSPR effects, but we showed that the perovskite crystallization dynamics was modified when adding the NP, leading to larger grains and a smoother surface, improving light absorption.

### 5.5 Conclusions

We considered only gold NPs in this section as they provide the best spectral matching with PSC needs. The NP volume fraction is key to determine the amount of additional light absorption by the active layer. In order to induce significant improvement, the volume fraction should be higher than 1%, which may not be easy to achieve experimentally while preserving the perovskite structural qualities. The gain on *J_sc_* is expected to be a only a few percent for a PSC with standard 300–400 nm MAPI thickness, because its absorption is already optimal, as discussed in section II. For such a structure, NP doping is not a useful strategy. On the other hand, if for various reasons the active layer thickness is limited to 100 or 150 nm, a 3% AuNP volume fraction increases light absorption by around 20%, of which half is useful, so that around 10 % gain in photovoltaic efficiency can be expected. The other half is dissipated as heat in the NP, therefore thermal risks should be taken into account.

Experimentally, enhancement of light absorption is manifested unambiguously by the shape of the EQE spectrum: it increases mostly in the red domain (type II behaviour in Figure 9). Based on this criterion, we found that only a few reports on PSC PCE increase can be attributed to enhanced light absorption. Because the NP volume fraction and the active layer thickness are generally not documented, it is impossible to say whether LSPR effects actually contribute, or whether the NPs favor light absorption indirectly by increasing the perovskite thickness or structural quality.

## 6 FINAL CONCLUSIONS

We have presented a panel of available theoretical methods, beyond FDTD, to describe the optical effect of a metallic nanoparticle in a perovskite solar cell. We can draw important conclusions which guide the understanding of the experimental state of the art as well as future works:

‐Simple analytical equations can predict the LSPR wavelength for NPs of a few nanometres, while Mie theory can be used for larger particles. Importantly, because the perovskite's index is usually very high, the LSPR wavelength in the device will be very different from the LSPR wavelength measured in solution. For gold NPs, the LSPR wavelength is conveniently located in the red domain (650 nm) where MAPI absorption needs to be improved. It is less interesting for silver NPs (589 nm), although these particles undergo less heat losses. Therefore, gold NPs appear as the most suited candidate for LSPR strategies in PSCs.
‐When the gold NPs are placed inside the MAPI layer, their effect on light absorption is significant for volume fractions of the order of 1% and above. Half of the additional light absorption due to the NP corresponds to enhanced light absorption by the perovskite around the NP and half to light absorption (and dissipation) by the NP itself.
‐Improvement of light absorption depends crucially on the thickness of the active layer: if the perovskite absorption is already optimal (thickness 300–400 nm for MAPI), a negligible gain can be expected by adding NP. For a lower thickness such as 100–150 nm, around 10% gain can be anticipated.
‐LSPR gain can thus be expected only for gold NP volume fractions of at least 1% and MAPI thickness no more than 100–200 nm. It is crucial that experimental reports provide the perovskite layer thickness and check that it is not modified by the NP addition, and estimate also the NP volume fraction.
‐Experimentally, the distinction between type I and type II EQE improvements provides a clear distinction between electrical gains and light absorption enhancement. The latter case is less common, and its origin is not clear. Quantitative optical modelling including LSPR near‐field and scattering have not been reported to match experimental data. Effects of the NPs on the perovskite's crystalline quality are a good alternative hypothesis to explain the improved light absorption.

## CONFLICT OF INTEREST STATEMENT

The authors declare no conflicts of interest.

## Supporting information

Supporting Information

## Data Availability

All data needed to evaluate the conclusions in the paper are present in the paper and/or in the Supporting Information. Additional data related to this paper may be requested from the authors.
