# Supplementary material for: Models of light absorption enhancement in perovskite solar cells by plasmonic nanoparticles
Source: Exploration (Beijing). 2023 Sep 6;4(1):20220146. doi: 10.1002/EXP.20220146 (PMC10867376; doi:10.1002/EXP.20220146)
Supplement: Supplementary file 1 — Supporting Information [file EXP2-4-20220146-s001.docx]

**Models of light absorption enhancement in perovskite solar cells by plasmonic nanoparticles**

Daming Zheng^a,b^, Thierry Pauporté^b^, Catherine Schwob^a^ and Laurent Coolen^a*^

*^a^ Sorbonne Université, CNRS, Institut de NanoSciences de Paris, INSP, F-75005, Paris, France.*

*^b^ Chimie ParisTech, PSL Research University, CNRS, Institut de Recherche de Chimie Paris (IRCP), UMR8247, 11 rue P. et M. Curie, F-75005 Paris, France.*

**SUPPORTING INFORMATION**

Figure S1: complex optical indices used in the calculations (blue : real component, green : imaginary component).

**A - Numerical absorption calculation**

We describe here the ratio between additional absorption in MAPI caused by the NP (useful absorption) and absorption by the NP (additional losses).

**Definitions :**

We used the software Lumerical to model a gold NP of radius 7 nm centered in a 100x100x100 nm^3^ homogeneous volume $\mathcal{V}$ of MAPI, with an incident plane wave from the top of the cell and phase-matching layers on the sides. The absorption within a given volume is calculated (in arbitrary units) as

$$A_{V}= \iiint_{V} \left\| \vec{E} \right\|^{2}\varepsilon^{''}dV$$

($\varepsilon''$ being the imaginary component of the local dielectric permittivity). The additional absorption induced by the presence of the NP is expressed as

$$\alpha= \frac{A_{\mathcal{V}}}{A_{\mathcal{V,}ref}}$$

where $A_{\mathcal{V}}$ is integrated over the whole volume of the simulation and $A_{\mathcal{V,}ref}$ is integrated over a reference volume containing only MAPI and no NP.

The additional absorption can be decomposed into $\alpha_{NP}+\alpha_{MAPI}$ with :

$$\alpha_{NP}= \frac{A_{NP}}{A_{\mathcal{V,}ref}} \alpha_{MAPI}=\frac{A_{MAPI}}{A_{\mathcal{V,}ref}}$$

where $A_{NP}$ and $A_{MAPI}$ are integrated respectively over the volumes of the NP and of MAPI (so that $A_{\mathcal{V}}= A_{NP}+A_{MAPI}$).

Eventually, the useful portion of the additional absorption caused by the NP is :

$$\eta= \frac{\alpha_{MAPI}}{\alpha}$$

**Gold 7-nm particle**

In order to model the whole volume of the LSP mode while achieving sufficient resolution near the metal-MAPI interface, we used a 0.25-nm mesh grid in the 20x20x20 nm^3^ sub-volume containing the NP and a 2-nm mesh in the rest of the simulation volume.

An important part of the simulation work by the software is to define the dielectric constant at each point, especially at the interface between materials : an average between the constants of the two materials is then taken in order to optimize the description of the interface. Here, because the gold index is 0.85 +i.1.82 at 500-nm wavelength and the MAPI index is 2.64 +i.0.63, we define as the volume of the NP all the points where the imaginary index component $n''$ at 500 nm is higher than 1.1 and as the outside volume all points where $n^{''}<1.1$.

We obtain the following raw values :

| Wavelength | 500 nm | 640 nm |
| --- | --- | --- |
| $A_{NP}$ | 0.454 | 3.70 |
| $A_{MAPI}$ | 153.2 | 97.44 |
| $A_{\mathcal{V,}ref}$ | 153.5 | 93.63 |

For the reference sample, the absorption is significantly higher at 500 nm than at 640 nm because of the stronger MAPI absorption. Upon adding the NP, the additional absorption by both gold and MAPI is much stronger at 640 nm due to the LSPR wavelength, and negligible at 500 nm.

From these values we can calculate at 640 nm the ratios :

| Wavelength | 640 nm |
| --- | --- |
| $\alpha_{NP}$ | + 3.95 % |
| $\alpha_{MAPI}$ | + 4.07 % |
| $\eta$ | 51 % |

**We conclude that half of the additional absorption occurs in MAPI (active layer) and half in gold (ohmic losses).**

**Gold 7-nm particle : definition of Au/MAPI boundary**

In the calculation above, the limit between the NP volume and the outside volume was set by the criterion $n^{''}=1.1$. We compare below the values of $\alpha_{NP}$, $\alpha_{MAPI}$ and $\eta$ obtained at 640 nm with a few different criteria ($A_{\mathcal{V}}$ and $A_{\mathcal{V,}ref}$ being unchanged for all choices) :

|  | $n^{''}=1.$1 | $n^{''}=0.8$ | $n^{''}=1.4$ | $n^{'}=1.2$ | $n^{'}=1.8$ | $n^{'}=2.4$ |
| --- | --- | --- | --- | --- | --- | --- |
| $\alpha_{NP}$ | 3.95 % | 4.04 % | 3.84 % | 3.35 % | 3.74 % | 4.03 % |
| $\alpha_{MAPI}$ | 4.07 % | 3.98 % | 4.19 % | 4.68 % | 4.01 % | 4.00 % |
| $\eta$ | 51 % | 50 % | 52 % | 58 % | 52 % | 50 % |

Using a criterion based on the real index rather than the imaginary index appears to give more uncertainty. This is because 1.7 % of the pixels in the simulated volume present an intermediate value (between 1.2 and 2.4) of the real index, while only 0.15 % of the pixels present an intermediate imaginary index (between 0.8 and 1.4). The boundary at the NP interface is sharper for the imaginary index than for the real index, so that changing the criterion on $n''$ leads to negligible change on $\eta$.

**Gold 7-nm particle : choice of the mesh size**

We now compare the absorptions calculated at 640 nm with different values of the mesh size in the 20x20x20 nm^3^ central sub-volume (with the criterion $n^{''}=1.1$):

| Mesh size | 2 nm | 1 nm | 0.5 nm | 0.25 nm |
| --- | --- | --- | --- | --- |
| $A_{NP}$ | 2.11 | 4.50 | 3.82 | 3.70 |
| $A_{MAPI}$ | 94.93 | 96.12 | 96.84 | 97.44 |
| $A_{\mathcal{V,}ref}$ | 93.76 | 93.77 | 93.68 | 93.63 |
| $\alpha_{NP}$ | 2.25 % | 4.80 % | 4.08 % | 3.95 % |
| $\alpha_{MAPI}$ | 1.25 % | 2.51 % | 3.37 % | 4.07 % |
| $\eta$ | 36 % | 34 % | 46 % | 51 % |

The value of $\eta$ thus depends crucially on the choice of mesh size, as a result of the difficulty to model adequately the gold/MAPI interface. We compare below the obtained $\eta$ for different criteria for the gold/MAPI interface and different mesh sizes :

|  | $n^{''}=1.$1 | $n^{''}=0.8$ | $n^{''}=1.4$ | $n^{'}=1.2$ | $n^{'}=1.8$ | $n^{'}=2.4$ |
| --- | --- | --- | --- | --- | --- | --- |
| mesh 2 nm | 36 % | 27 % | 43 % | 69 % | 43 % | 33 % |
| mesh 1 nm | 34 % | 33 % | 39 % | 69 % | 40 % | 32 % |
| mesh 0.5 nm | 46 % | 44 % | 47 % | 62 % | 50 % | 47 % |
| mesh 0.25 nm | 51 % | 50 % | 52 % | 58 % | 52 % | 50 % |

For the larger mesh sizes, the value of $\eta$ depends very strongly on the criterion for definition of the interface, showing that the precision of the simulation is insufficient. For 0.25-nm mesh size, on the other hand, we can consider that we have converged to an accurate result with around 5 % precision.

**Silver 7-nm particle**

We now replace gold by silver for the 7-nm particle, use a 0.25-nm mesh size for the central sub-volume and consider the LSPR wavelength 589 nm. We find the following values, depending on the criterion chosen for the silver/MAPI interface (with $n_{Ag}$ = XXX at 589 nm).

|  | $n^{''}=0.6$ | $n^{''}=2.1$ | $n^{''}=3.6$ | $n^{'}=0.3$ | $n^{'}=1.3$ | $n^{'}=2.3$ |
| --- | --- | --- | --- | --- | --- | --- |
| $\alpha_{NP}$ | + 1.7 % | + 1.6 % | + 1.5 % | 1.1 % | 1.3 % | 1.5 % |
| $\alpha_{MAPI}$ | + 4.1 % | + 4.2 % | + 4.4 % | 4.7 % | 4.5 % | 4.3 % |
| $\eta$ | 70 % | 72 % | 75 % | 81 % | 77 % | 74 % |

The 0.25-nm mesh size can thus be considered sufficient to state that $\boldsymbol{\eta}$ **= 70-80 % of the additional absorption corresponds to useful absorption.**

**Gold particles of different sizes**

The same protocol was reproduced for gold nanoparticles of different radii : 3 and 15 nm. The mesh size was 2 nm, except in the 20x20x20 nm^3^ central volume where it was 0.25 nm in the former case and 1 nm in the latter.

| NP radius | 7 nm | 3 nm | 15 nm |
| --- | --- | --- | --- |
| $\alpha_{NP}$ | + 3.95 % | + 0.3 % | + 25 % |
| $\alpha_{MAPI}$ | + 4.07 % | + 0.25 % | + 22 % |
| $\eta$ | 51 % | 45 % | 45 % |

The increase from 3 to 7 nm is proportional to the NP volume increase, as expected from the expression of $\sigma_{abs}$ in the small-NP regime. On the other hand, the increase from 7 to 15 nm is less than the volume increase, indicating a departure from the small-NP regime (consistent with Mie theory : fig. 6). **For all NP sizes considered, around half of the absorption increase corresponded to losses in the NP and half to useful MAPI absorption.**

**B – LSPR properties obtained by Mie theory**

Figure S2: spectrum of the absorption and scattering efficiency factors $Q_{abs}$ and $Q_{sca}$ for a spherical Au sphere as a function of its radius in water, glass, FTO, TiO_2_ and Spiro-OMeTAD, as given by Mie theory.

Figure S3: spectrum of the absorption and scattering efficiency factors $Q_{abs}$ and $Q_{sca}$ for a spherical Ag sphere as a function of its radius in water, glass, FTO, TiO_2_ and Spiro-OMeTAD, as given by Mie theory.

Figure S4: spectrum of the absorption and scattering efficiency factors $Q_{abs}$ and $Q_{sca}$ for a spherical Al sphere as a function of its radius in water, glass, FTO, TiO_2_ and Spiro-OMeTAD, as given by Mie theory.

Figure S5: spectrum of the absorption and scattering efficiency factors $Q_{abs}$ (full line) and $Q_{sca}$ (dotted line) for a spherical silver (left) and aluminum (right) sphere in water, for 4 different sphere radii, as given by Mie theory. For the AgNP $R=$2 nm, the absorption and scattering efficiencies are multiplied by 2 and 1000 respectively for clarity.

Figure S6: spectrum of the absorption and scattering efficiency factors $Q_{abs}$ (full line) and $Q_{sca}$ (dotted line) for a spherical silver (left) and aluminum (right) sphere in MAPI, for 4 different sphere radii, as given by the generalized Mie theory.

Figure S7: spectrum of the absorption and scattering efficiency factors $Q_{abs}$ (full line) and $Q_{sca}$ (dotted line) for a spherical gold sphere in TiO_2_ (left) and spiro-OMeTAD (right), for 4 different sphere radii, as given by Mie theory.

Figure S8: scattering diagram (normalized) of a gold nanosphere of radius 60 nm in TiO2 (left) and spiro-OMeTAD (right) at various wavelengths, as calculated by Mie theory (neglecting the imaginary component in the Spiro-OMeTAD optical index).

Figure S9: scattering diagram (norm.) of a gold nanosphere in water, as calculated by Mie theory, (a) at the LSPR wavelength (wavelength of maximal absorption efficiency factor) for various radii, (b) at various wavelengths for a 60-nm radius.

**C – Experimental state of the art : photovoltaic performance**

Figure S10 plots the distribution of photovoltaic PSC properties reported in the various articles of the literature. Each dot corresponds to an experimental paper, with different colors and shapes for different positions within the PSC. Some authors compared different device parameters (such as NP geometry or concentration) : we report only the results for the most efficient device.

Figure S10(a) plots the obtained power conversion efficiency (PCE) for the PSC with metallic NPs as a function of the PCE for the reference device (without NPs). Although all papers report a PCE improvement due to the metallic NPs (which might be a result of the bias introduced by the difficulty to publish negative results in the scientific community), some of these improvements are minor. The best relative improvements are by a factor 1.3 to 1.6. They are for cases with relatively low PCE in the reference device, showing that the NP addition process may have corrected some of the defects of the PSC crystalline structure. However, for cells of originally good quality, the nanoparticles still produced a significant PCE improvement : we found on average, for the devices with reference PCE above 15 %, a 2 % absolute increase ($PCE- {PCE}_{ref}$) and a 12 % relative increase ($\Delta PCE= \left( PCE- {PCE}_{ref} \right)/{PCE}_{ref}$).

Figures S10(b) and (c) plot respectively the short-circuit current ($J_{sc}$) and the open-circuit voltage ($V_{oc}$) of the improved device as a function of the value for the reference device. On average, the short-circuit current was increased by an absolute + 1.7 mA/cm² (relative improvement of 9 %) while the open-circuit voltage was increased by + 30 mV (relative improvement of 3 %). Again, the strongest improvements were obtained for quantities which were originally low in the reference device. For the higher reference values of $J_{sc}$, the NPs still generated a significant improvement (average relative + 7 % increase for reference current above 19 mA/cm²). On the other hand, for the reference devices with the highest $V_{oc}$, the NPs allowed almost no gain (+ 1.7 % relative increase for reference voltage above 1.05 V).

Finally, figures S10(d) and (e) plot respectively the relative improvements $\Delta J_{sc}$ and $\Delta V_{oc}$ as a function of $\Delta PCE$. The 3 devices with strongest PCE improvement ($\Delta PCE>$40 % - these correspond to reference devices with low PCE) were associated with a significant increase of $V_{oc}$. For the rest of the reports, stronger PCE increases appear related to stronger improvements of $J_{sc}$, while there is little correlation between $\Delta PCE$ and $\Delta V_{oc}$. We also found little correlation between the PCE improvement and a change in the fill factor.

This short-circuit current improvement was attributed by some authors to a better light absorption, due to either near-field of scattering mechanisms [21,24,27,31,32,37,41,44,45,56]. However, many also mention the possibility to electrical effects [12,14,18,19,22,23,25,26,28,33,35, 42,43,46,49,53,57,58]. Possible electrical effects are transport and collection improvements, suppression of charge recombination channels, and a better exciton dissociation as demonstrated in ref. [27]. In some papers [15,17,20,30,36,38,39,47,48,50], the efficiency improvement was attributed only to electrical effects, with no or very little absorption enhancement.

**NP position:** the choice of the NP position is of course crucial to their effect on the PSC efficiency but is limited by the difficulty to introduce the NPs to the deposition protocol without disturbing the cell’s crystalline quality. Several groups have introduced the NPs directly within the perovskite layer [27-29] or at the perovskite/ETL [30-35] or perovskite/HTL [21-26] interface, so that the NPs are sufficiently close to the perovskite to act on its absorption by near-field effects. Most reports present NPs in the ETL [36-58], in the HTL [15-20] or at the HTL/electrode interface [13,14].

The curves in fig. S10 show no significant dependence on the position of the NP inside the device : although this is a key parameter, its role seems obscured by the many other relevant parameters (NP composition, shape and concentration, device overall crystalline quality etc) which differ between different works.

**Conclusion:** there is a broad range of experimental results for the introduction of metallic NPs in a PSC. The most striking improvements of the PCE, $J_{sc}$ or $V_{oc}$ were obtained when these values were originally relatively low in the reference device : in these cases, it seems plausible that the role of the NPs was mainly to help mitigate the formation of structural defects in the device (crystalline structure, grains and boundaries etc).

On the other hand, for the reference devices with relatively higher efficiency, some improvement of the PCE could still be obtained (around + 2 % in absolute value). It was associated with an improvement of $J_{sc}$, while changes on $V_{oc}$ and in the fill factor were lower and showed little correlation with the PCE improvement.

Figure S10 : (a) PCE of the improved PSC (containing metallic NPs) as a function of the PCE original reference PSC (without NPs). The given PCEs are for the champion device of each configuration. (b) Short-circuit current of the improved PSC (with NPs) as a function of the reference current. (c) Open-circuit voltage of the improved PSC as a function of the reference voltage. (d) Relative short-circuit current improvement ${\Delta J}_{sc}=(J_{sc}-J_{sc,ref})/J_{sc,ref}$ as a function of the relative PCE improvement. (e) Relative open-circuit improvement as a function of the relative PCE improvement.
